# Supplementary material for: The Loss of Gonadal Hormones Has a Different Impact on Aging Female and Male Mice Submitted to Heart Failure-Inducing Metabolic Hypertensive Stress
Source: Cells. 2025 Jun 9;14(12):870. doi: 10.3390/cells14120870 (PMC12190614; doi:10.3390/cells14120870)
Supplement: Supplementary file 1 [file cells-14-00870-s001.zip › cells-3650924-supplementary.pdf]

SUPPLEMENTAL DATA

The loss of gonadal hormones has a different impact on aging female and male mice submitted to heart failure-inducing metabolic-hypertensive stress.

Diwaba Carmel Teou, Élisabeth Walsh-Wilkinson, Emylie-Ann Labbé, Sara-Ève Thibodeau, Audrey Morin-Grandmont, Ann-Sarah Trudeau, Marie Arsenault, Jacques Couet*

Groupe de recherche sur les valvulopathies, Centre de recherche de l'Institut universitaire de cardiologie et de pneumologie de Québec, Université Laval, Québec City, Québec, Canada

*: Correspondence to jacques.couet@med.ulaval.ca

Table S1. Sequences of primers used in this study.

Symbol	Description	Forward sequence<br>Reverse sequence
Bdh1	3-Hydroxybutyrate Dehydrogenase 1	5'-AGG ACA AAG GTG ATG CTG GG-3'<br>5'-CCA AAC GTT GAG ATG CCT GC-3'
Col1a1	Collagen Type I Alpha 1 Chain	5'-CAT TGT GTA TGC AGC TGA CTT C-3'<br>5'CGC AAA CAC TCT ACA TGT CTA GG-3'
Col3a1	Collagen Type III Alpha 1 Chain	5'-TCT CTA GAC TCA TAG GAC TGA CC-3'<br>5' TTC TTC TCA CCC TTC TTC ATC C-3'
Fat/Cd36	Fatty Acid Translocase/CD36	5'-GAT CGG AAC TGT GGG CTC AT-3'<br>5'-CTT TGC CAC GTC ATC TGG G 3'
Glut4	Glucose Transporter Type 4	5'-CAG TAC TCC CTG CTC TCC TG-3'<br>5'-GCT CTC TCT CCA ACT TCC GT-3'
Nppa	Natriuretic Peptide B	5'-CTC CTT GGC TGT TAT CTT CGG-3'<br>5'-GGG TAG GAT TGA CAG GAT TGG-3'
Nppb	Natriuretic Peptide A	5'-AGG TGA CAC ATA TCT CAA GCT G-3'<br>5'-CTT CCT ACA ACA TCA GTG C-3'
Pdk4	Pyruvate Dehydrogenase Kinase 4	5'-TTC TTG AAG AGT TCG AAG AGC A-3'<br>5'-CAT CGC CAG AAT TAA ACC TCA C-3'
Ppia	Cyclophilin a	5'-TTC ACC TTC CCA AAG ACC AC-3'<br>5'-CAA ACA CAA ACG GTT CCC AG-3'
Postn	Periostin	5'-GCT TTC GAG AAA CTG CCA CG-3'<br>5'-ATG GTC TCA AAC ACG GCT CC-3'
Thbs4	Thrombospondin 4	5'-GAT ACT GAC GGG GAT GGG AG-3'<br>5'-CGT CAC TGT CTT GGT TGG TG-3'

Table S2. Echocardiography data. EDV: end-diastolic volume, ESV: end-systolic volume, HR: heart rate, SV: stroke volume, CO: cardiac output and bpm: beats per minute. Data are represented as mean +SEM. Two-way ANOVA followed by Holm-Sidak post-test. a : $p<0.05$. b : $p<0.01$. c : $p<0.001$ and d : $p<0.0001$, Controls vs Gonadactomized animals (Gx). α : $p<0.05$. β : $p<0.01$. γ : $p<0.001$ and δ : $p<0.0001$, Young vs. Old mice.

**Males**	Controls		Gx		p-value Age	p-value Ocx
Paramètres	Young (n=8)	Old (n=8-10)	Young (n=8-10)	Old (n=8)		
EDV (µl)	53.8±3.08	75.0±4.01$^{\gamma}$	46.9±1.88	51.3±1.64^{d}	0.0004	<0.0001
ESV (µl)	23.1±0.89	34.9±2.45$^{\gamma}$	19.3±0.99	18.9±0.88^{d}	0.0035	<0.0001
HR (bpm)	498±10.4	471±12.5	523±22.5	502±12.7	0.12	0.074
SV (uL)	30.7±2.24	40.1±2.22$^{\beta}$	27.7±1.35	32.5±1.19$^{\beta}$	0.0011	0.011
CO (ml/min)	15.3±1.06	18.9±1.20$^{\alpha}$	14.3±0.63	16.3±0.69	0.011	0.094
**Females**						
Parameters	Young (n=8)	Old (n=8-10)	Young (n=8-10)	Old (n=8)	p-value Age	p-value Ovx
EDV (µl)	47.0±3.03	61.9±2.46$^{\gamma}$	42.5±2.32	56.1±2.58$^{\gamma}$	<0.0001	0.059
ESV (µl)	17.1±1.51	25.1±1.56$^{\gamma}$	16.6±1.12	22.1±1.00$^{\beta}$	<0.0001	0.21
HR (bpm)	516±11.2	480±32.1	505±11.9	529±16.5	0.77	0.31
SV (uL)	31.5±1.67	34.9±2.31	25.9±1.28^{b}	35.7±1.33$^{\delta}$	<0.0001	0.067
CO (ml/min)	16.1±0.55	16.7±1.88	12.2±0.33^{a}	17.8±0.99$^{\gamma}$	0.0069	0.20

Table S3. Echocardiography data. A': A' wave velocity, IVRT: isovolumetric relaxation time, MPI: myocardial performance index, LA: left atrial Two-way ANOVA followed by Holm-Sidak post-test. a : $p<0.05$. b : $p<0.01$. c : $p<0.001$ and d : $p<0.0001$, Controls vs Gonadactomized animals (Gx). α : $p<0.05$. β : $p<0.01$. γ : $p<0.001$ and δ : $p<0.0001$, Young vs. Old mice.

**Males**	Controls		Gx		p-value Age	p-value Ocx
Parameters	Young (n=8)	Old (n=8-10)	Young (n=8-10)	Old (n=8)		
A' (mm/s)	-17.8±0.81	-18.7±1.44	-14.2±0.45	-20.4±1.19$^{\gamma}$	0.0060	0.43
IVRT (ms)	16.2±0.53	15.6±0.64	17.3±0.66	15.7±0.54	0.067	0.38
E'/A'	1.66±0.038	1.59±0.071	1.95±0.042^{b}	1.54±0.036$^{\gamma}$	0.0001	0.036
MPI	0.67±0.023	0.66±0.019	0.67±0.026	0.68±0.016	0.76	0.99
LA diam. (mm)	2.34±0.049	2.50±0.071	2.17±0.037^{a}	2.38±0.062	0.014	0.19
**Females**						
Parameters	Young (n=8)	Old (n=8-10)	Young (n=8-10)	Old (n=8)	p-value Age	p-value Ovx
A' (mm/s)	-18,8±0,56	-19,5±2,02	-19,0±0,65	-19,6±0,92	0,59	0,89
IVRT (ms)	15,1±0,29	16,8±1,27	16,1±0,60	15,0±0,33	0,67	0,55
E'/A'	1,70±0,0038	1,56±0,096	1,64±0,039	1,64±0,055	0,23	0,92
MPI	0,69±0,027	0,64±0,019	0,65±0,028	0,66±0,021	0,41	0,75
LA diam. (mm)	2,13±0,026	2,36±0,076$^{\beta}$	2,10±0,047	2,31±0,041$^{\beta}$	0,0001	0,40

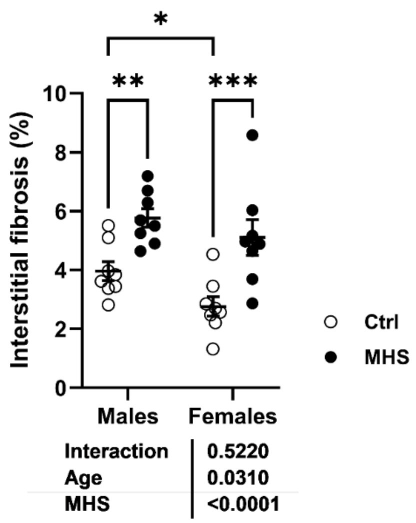

Figure S1. Myocardial interstitial fibrosis after MHS in young mice. Data are represented as mean ± SEM (n=8 per group). Two-way ANOVA followed by Holm-Sidak post-test. *: p<0.05, **: p<0.01, and ***: p<0.001 between indicated groups.

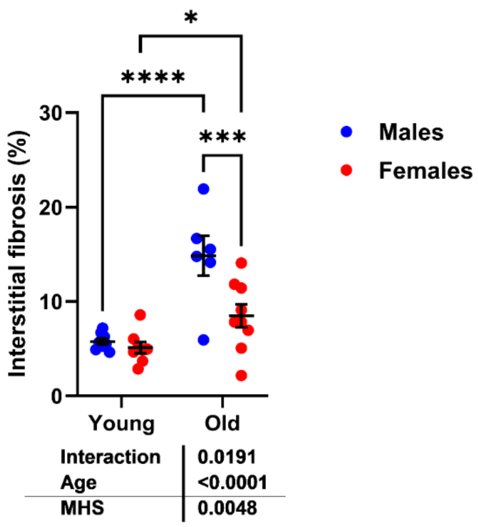

Figure S2. Myocardial interstitial fibrosis after MHS in old intact mice. Data are represented as mean ± SEM (n=8 per group). Two-way ANOVA followed by Holm-Sidak post-test. *: p<0.05, **: p<0.01, and ***: p<0.001 between indicated groups.

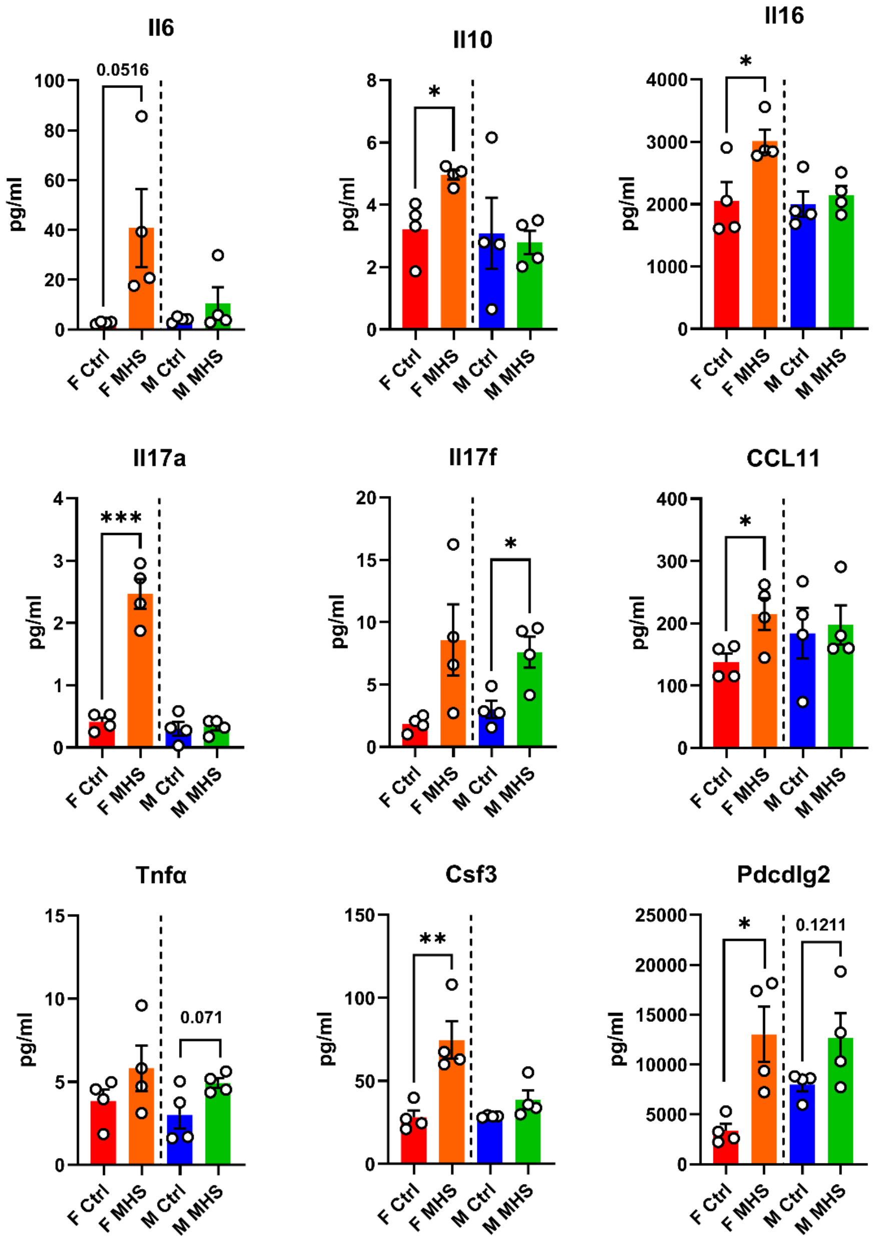

Figure S3. Plasma protein concentration of 9 immunity-related molecules in young male (M) and female (F) mice exposed or not to MHS. Levels of these molecules were evaluated as described in the Materials and Methods section. Il-6: interleukin-6, Il-10: interleukin-10, Il-16: interleukin-16, IL-17: interleukin 17, CCL11: C-C Motif Chemokine Ligand 11, Tnfα: tumour necrosis factor alpha, Csf3: Colony Stimulating Factor 3, and Pdcd1lg2: Programmed Cell Death 1 Ligand 2. Data are represented as mean ± SEM (n=4). Student T-test between young and old animals. *: $p<0.05$, **: $p<0.01$ and, ***: $p<0.001$ and ****: $p<0.0001$ between indicated groups.

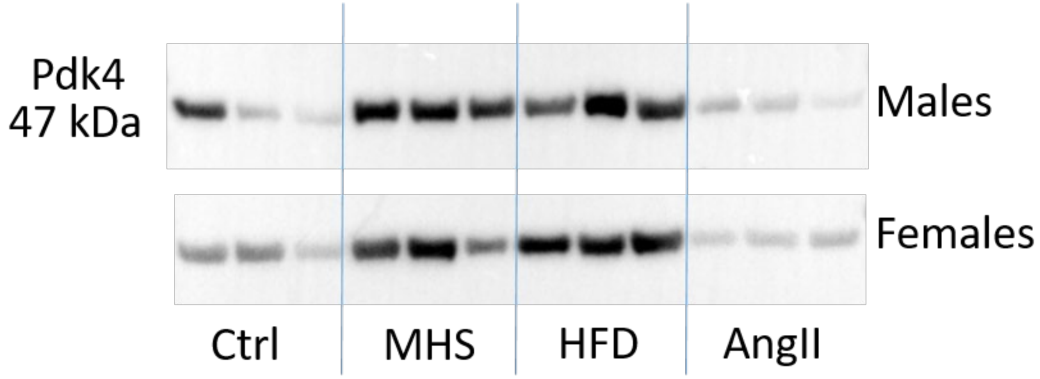

Figure S4. The HFD is responsible for the myocardial PDK4 protein content after MHS. Young male and female mice were administered the MHS treatment for 28 days, or the HFD and AngII alone.
